# Bisphenol A Interferes with Mast Cell-Mediated Promotion of Cellular Processes Critical for Spiral Artery Remodeling

**DOI:** 10.3390/ijms26199706

**Published:** 2025-10-05

**Authors:** Federica Romanelli, Ningjuan Zhang, Mario Bauer, Beate Fink, Ana Claudia Zenclussen, Anne Schumacher, Nicole Meyer

**Affiliations:** 1 Department of Environmental Immunology, Helmholtz Centre for Environmental Research Leipzig-Halle UFZ, 04318 Leipzig, Germany; federica.romanelli@ufz.de (F.R.); n.zhang@klinikum-oberggoelzsch.de (N.Z.); mario.bauer@ufz.de (M.B.); beate.fink@ufz.de (B.F.); ana.zenclussen@ufz.de (A.C.Z.); anne.schumacher@ufz.de (A.S.); 2 Perinatal Immunology, Saxon Incubator for Clinical Translation (SIKT), Medical Faculty, University Leipzig, 04103 Leipzig, Germany

**Keywords:** bisphenol A, mast cells, spiral artery remodeling

## Abstract

Mast cells (MCs) belong to the cell network that regulates uterine spiral artery remodeling (uSAR), a critical vascular adaptation supporting placental development and fetal growth. Our previous in vitro study demonstrated that human MCs promote trophoblast invasion, as well as uterine vascular smooth muscle cells (uVSMCs) migration and transition to a synthetic phenotype—essential steps for a successful uSAR. Although MCs are known targets of bisphenol A (BPA), a widespread endocrine-disrupting chemical, its impact on their supportive role in uSAR is unknown. In this study, we used murine cell lines to investigate whether BPA (0.1–100 µM) affects MC-mediated promotion of cellular processes critical for uSAR. Our results showed that BPA exposure hindered MCs’ ability to promote trophoblast invasion and the switch in uVSMCs’ synthetic phenotype and migration. The highest concentrations of BPA altered the expression of genes related to MCs activation and proliferation, and of those involved in trophoblasts invasion. In contrast, low doses induced the expression of pro-inflammatory mediators in MCs without detectable effect on trophoblasts at the transcriptional level. These findings confirmed MCs as key mediators of uSAR, and identified BPA as a disruptor of their function, emphasizing its potential harmful impact on reproductive health.

## 1. Introduction

The successful progression of pregnancy strongly depends on the proper activity of hormones, which coordinate the complex and dynamic network of maternal and fetal cells throughout gestation [1,2]. For this reason, exposure to endocrine-disrupting chemicals (EDCs) represents a huge concern for women’s reproductive health [3]. EDCs are exogenous chemicals capable of interfering with any aspect of hormonal regulation, potentially threatening the fine-tuned control of the placenta and fetal development [4,5,6]. The Endocrine Society reported that approximately 1000 EDCs are present in our environment, primarily originating from manufacturing processes and commonly used in products such as cosmetics, pesticides, food packaging, toys, and more [7].

Bisphenol A (BPA) is one of the best studied EDCs and is used in high quantities in the production of polycarbonate plastics and epoxy resins. Human exposure to BPA occurs via ingestion, inhalation, or dermal contact [8]. A systematic review and meta-analysis comprising 15 population-based studies across six continents showed that BPA was detectable in the urine of over 90% of participants, highlighting the ubiquitous exposure to this chemical in everyday life [9]. Despite being rapidly metabolized and excreted through urine, BPA is found in human blood, breast milk, amniotic fluid, and the placenta. Indeed, there is substantial evidence showing that BPA can cross the maternal–fetal barrier, reaching fetal tissues and blood [10,11,12,13]. Once in the tissue, BPA has the potential to interfere with physiological endocrine signaling, as its structure allows it to bind to or interact with a multitude of receptors, such as estrogen receptor (ER) α, ERβ, G-protein coupled estrogen receptor (GPER), and estrogen-related receptor γ (ERRγ) [14,15,16]. Given that placental and fetal development rely critically on the precise regulation of hormonal signaling, these findings are particularly concerning [17,18]. Accordingly, numerous in vitro and in vivo studies have investigated the impact of prenatal BPA exposure on placental development and pregnancy outcomes [6,19]. Despite the existence of controversial results—primarily due to differences in BPA concentrations, exposure times, various models used, and experimental setups—more and more findings support the harmful impact of this chemical on reproductive processes [20,21]. Indeed, numerous studies have shown that BPA exposure could interfere with women’s reproductive health as early as during ovulation, hindering the proper maturation of oocytes, mainly by causing meiotic abnormalities [22,23,24]. Moreover, higher BPA levels in the urine or blood have been associated with reduced oocyte retrieval and implantation failure, as well as with an increased risk of polycystic ovarian syndrome and endometriosis in women [25,26,27]. Additionally, various studies have reported that exposure of pregnant mice to BPA during gestation and lactation can lead to transgenerational effects that persist across multiple generations. These effects are often sex-specific, affecting the male progeny more, and manifest as metabolic alterations characteristic of disorders such as obesity and diabetes. These long-lasting consequences seem to be mediated by epigenetic alterations in the germline of in utero-exposed fetuses, including DNA methylation, histone modifications, chromatin accessibility, and changes in non-coding RNA expression [28,29,30,31]. In 2018, our group showed that mice exposed during the first 7 days of gestation to 50 µg/kg BPA/day—at that time approved as tolerable daily intake (TDI) dose by the European Food Safety Authority (EFSA)-led to fetal growth restriction (26.2% of the litter), significant smaller placental areas, and impaired uterine spiral artery remodeling (uSAR) [32]. uSAR is a crucial physiological process in which spiral arteries (SAs), the terminal branches of the uterine arteries, are restructured to enhance vascular capacity while reducing vascular resistance [33]. This remodeling is a vital maternal adaptation to the increased blood volume directed to the uterus and ensures a steady, low-pressure blood flow to the placenta and an efficient exchange of the essential nutrients, gases, and waste products between the mother and the fetus [34]. Impaired uSAR has been associated with adverse outcomes for both maternal and fetal health, including preeclampsia, fetal growth restriction, and pre-term labor [35,36,37,38,39]. Maternal vascular and immune cells, along with fetal extravillous trophoblast cells (EVTs), are key players in the well-characterized steps of the remodeling process, among which the invasion of EVTs in the uterine tissue and vessels, and the phenotype switch of uterine vascular smooth muscle cells (uVSMCs) are of fundamental importance [37]. In particular, uVSMCs transition from a contractile to a synthetic phenotype at the onset of the remodeling process, enabling their proliferation and migration out of the vessel, where they predominantly undergo apoptosis [40]. By mid-gestation, this process results in SAs with an enlarged lumen, a thinner wall containing EVTs encapsulated in a fibrinoid matrix, and reduced vascular resistance due to the loss of uVSMCs [41].

Although the importance of uSAR is undeniable, and concerns about BPA’s effects on reproductive processes continue to grow, a significant knowledge gap remains regarding the mechanisms through which BPA may interfere with this critical vascular adaptation. Unraveling these mechanisms presents significant challenges, not only due to the diverse modes of action and wide range of targets associated with BPA, but also because of the complex crosstalk of cells and mediators involved in the different stages of uSAR.

While for a long time uterine natural killer cells (uNKs) and macrophages were thought to be the main immune cell players involved in uSAR, we recently identified mast cells (MCs) as novel mediators of this process [42,43]. Using different mouse models characterized by MCs deficiency, we proved that the presence of these cells is required for an optimal uSAR process [44,45,46]. Subsequently, we began to explore possible pathways through which MCs may support uSAR. We discovered that human MCs are able to enhance EVT proliferation and invasiveness and to promote primary human uVSMCs phenotype switch and migration in vitro [47]. These results laid the foundations of the present study, which aims to elucidate whether MCs’ exposure to BPA interferes with their observed promotion of EVTs and uVSMCs functionality in the context of uSAR. While some studies have already reported the effects of BPA exposure on human MCs activation and degranulation, to the best of our knowledge, no studies have addressed its potential indirect effects on cells influenced by MCs activity [48,49,50]. As the activity of MCs and their recruitment in the uterus is influenced by estrogens, we hypothesize that the exposure to BPA could interfere with their key role in supporting uSAR-related cellular processes [51,52,53]. In line with our earlier study, we performed in vitro functional assays coculturing mouse MCs (MC/9 cell line) with invasive trophoblasts (SM9-2 cell line) or primary mouse uVSMCs. First, we confirmed MCs-mediated promotion of trophoblasts and uVSMCs functions related to key processes underlying uSAR in mouse cell cultures. Building on these findings, we then exposed the cells under the same experimental conditions to various concentrations of BPA (0.1, 1, 10, and 100 µM) to assess its potential direct and indirect effects on the observed MCs’ positive influence on trophoblasts and uVSMCs functionality.

## 2. Results

### 2.1. ERα Is Expressed in SM9-2 and MC/9 Cell Lines, as Well as in Primary uVSMCs

**Figure 1:**
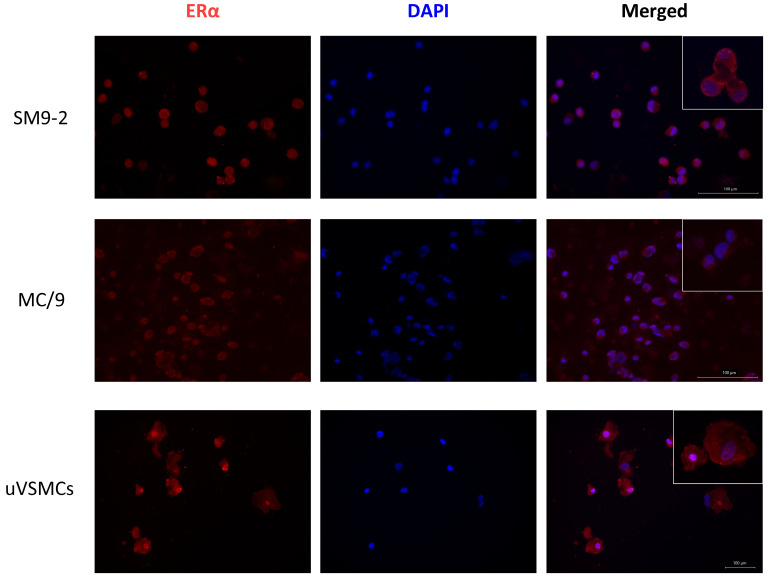
ERα is expressed in mouse SM9-2 and MC/9 cell lines, as well as in mouse primary uVSMCs. In the first column, ERα-related fluorescence is visible in red. The cell nuclei were stained with DAPI (second column, blue). In the third column, the merged fluorescent signals are shown, while in the upper right corner, a closer zoom on the cells is presented. ERα, estrogen receptor α; uVSMCs, uterine vascular smooth muscle cells.

The first step in assessing whether BPA impacts MCs-mediated promotion of trophoblasts and uVSMCs cellular functions was to confirm that the chosen cell lines express at least one of the potential binding sites for the chosen chemical. Therefore, ERα expression was analyzed in mouse MC/9 and SM9-2 cell lines, as well as in mouse primary uVSMCs, using immunocytochemistry. Our results showed that ERα was expressed in all selected cell lines and primary cells in the nuclei, cytoplasm, and on the cell membrane (Figure 1).

### 2.2. BPA Negatively Impacts MCs’ Ability to Promote SM9-2 Trophoblast Invasiveness

**Figure 2:**
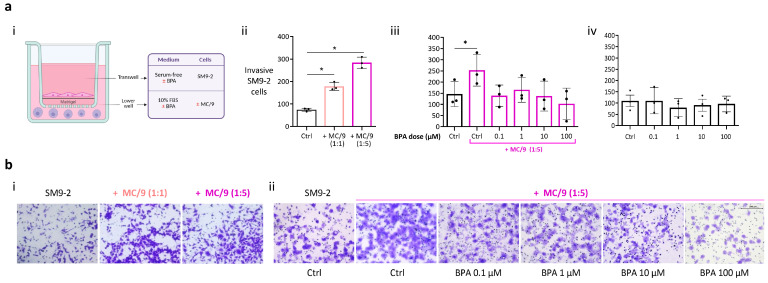
BPA exposure negatively affects the MC/9 cell ability to increase SM9-2 cell invasiveness, but does not directly impact trophoblasts’ invasive potential. (**a**) Graphical scheme ((**i**); created in BioRender, accessed on 23 April 2025, https://BioRender.com/akx5gxx) and results of the invasion assay with SM9-2 trophoblasts. The graphs illustrate the number of SM9-2 cells that invaded the matrigel layer after 24 h under different experimental conditions: cocultured with MC/9 cells ((**ii**); 1:1 and 1:5 cell-to-cell ratios; *n* = 3); exposed to BPA at different concentrations, either cultured with MC/9 cells ((**iii**); 1:5 cell-to-cell ratio; *n* = 3) or cultured alone ((**iv**); *n* = 3). Repetitive measures ANOVA was conducted, and all groups’ means were compared to each other using Tukey’s multiple comparison post hoc test. Statistical significance was defined as *p* < 0.05. (**b**) Representative images of crystal violet-stained SM9-2 cells on the lower side of the transwell membrane under different experimental conditions: cocultured with MC/9 cells ((**i**); 1:1 and 1:5 cell-to-cell ratios); exposed to BPA at different concentrations when cocultured with MC/9 cells ((**ii**); 1:5 cell-to-cell ratio). *p* value: * ≤ 0.05; BPA, bisphenol A; Ctrl, control (DMSO).

The degree and timing of trophoblast invasion into the maternal endometrial tissue and arteries are crucial in determining the fate of uSAR [54]. When SM9-2 trophoblasts were cocultured with MC/9 cells, trophoblast invasion was significantly enhanced (cell-to-cell ratios 1:1 and 1:5, *p* < 0.05) (Figure 2a(ii)). The same experiment was repeated, culturing SM9-2 cells with or without MC/9 cells (cell-to-cell ratio 1:5) in BPA-containing medium at different concentrations (0.1, 1, 10, 100 µM) for 24 h. The results showed that BPA exposure negatively impacted the invasion rate of trophoblasts co-cultured with MCs. However, the number of invading cells was not significantly lower compared to the untreated co-culture group. Nonetheless, the statistically significant increase in invasiveness observed when SM9-2 trophoblasts were co-cultured with MC/9 cells was lost when cells were exposed to BPA at any of the used concentrations (Figure 2a(iii)). On the other hand, the invasion rates of trophoblasts cultured alone were not impacted (Figure 2a(iv)). Representative pictures of the invasion assay are presented in Figure 2b.

### 2.3. BPA at High Dose Impacts the Proliferation and Viability of SM9-2 Cells

**Figure 3:**
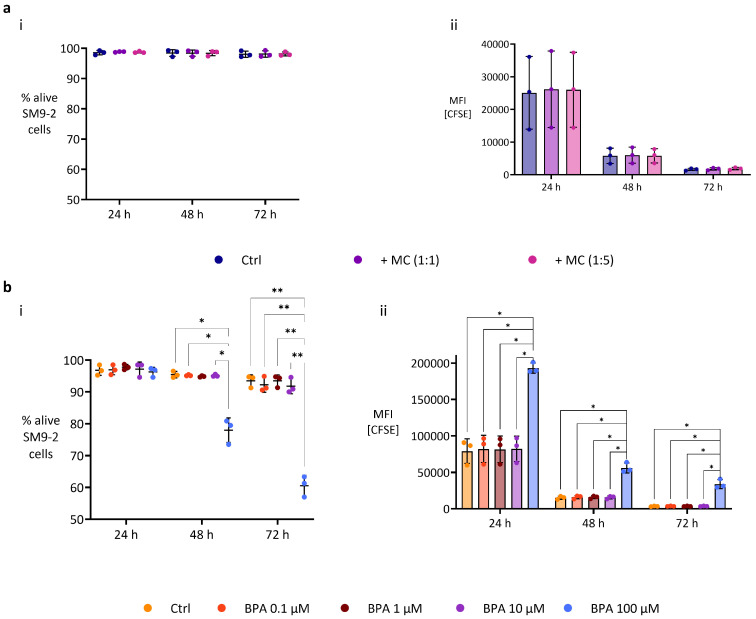
MC/9 cells do not affect the proliferation or viability of SM9-2 cells, whereas both are negatively impacted by BPA at a concentration of 100 µM. Graphs show the results of the CFSE-based proliferation (**i**) and viability (**ii**) assays when cocultured with MC/9 cells (**a**) or when exposed to different BPA concentrations (**b**) for 24 h. Proliferation was measured through the MFI of the CFSE dye, which decreases as cells proliferate. Viability was assessed using eBioscience™ Fixable Viability Dye. Two-way ANOVA was conducted, and all groups’ means were compared to each other using Tukey’s multiple comparison post hoc test. Statistical significance was defined as *p* < 0.05. *p* value: * ≤ 0.05; ** ≤ 0.01; BPA, bisphenol A; CFSE, carboxyfluorescein succinimidyl ester; MFI, median fluorescence intensity.

The precise regulation of trophoblast proliferation is critical for several stages of placental and embryonic development [55]. We determined whether MC/9 cells influence the viability and proliferation of SM9-2 trophoblasts. The presence of MC/9 cells had no effect on the viability or proliferation of SM9-2 (Figure 3a). Therefore, we only tested the direct effects of BPA exposure on trophoblast proliferation, as MCs’ presence did not influence SM9-2 cells’ proliferative behavior. We found that BPA at a concentration of 100 µM significantly inhibited the cells’ proliferation at all time points. However, lower BPA concentrations did not hinder SM9-2 cells’ proliferation (Figure 3b(i,ii)). Notably, the highest dose of BPA exhibited substantial cytotoxicity starting after 48 h of exposure, reducing the percentage of alive cells to barely 60% at 72 h (Figure 3b(i)). The precise regulation of trophoblast proliferation is critical for several stages of placental and embryonic development [40]. We determined whether MC/9 cells influence the viability and proliferation of SM9-2 trophoblasts. The presence of MC/9 cells had no effect on the viability or proliferation of SM9-2 (Figure 3a). Therefore, we further tested only the direct impact of BPA exposure on trophoblasts’ proliferation, as MC’s presence did not influence SM9-2 cells’ proliferative behavior. We found that BPA at a concentration of 100 µM significantly inhibited the cells’ proliferation at all time points. However, lower BPA concentrations did not hinder SM9-2 cells’ proliferation (Figure 3b(ii)). Notably, the highest dose of BPA exhibited substantial cytotoxicity starting after 48 h of exposure, reducing the percentage of alive cells to barely 60% at 72 h (Figure 3b(i)).

### 2.4. MCs-Mediated Promotion of uVSMCs’ Phenotype Switch Is Hindered by BPA

**Figure 4:**
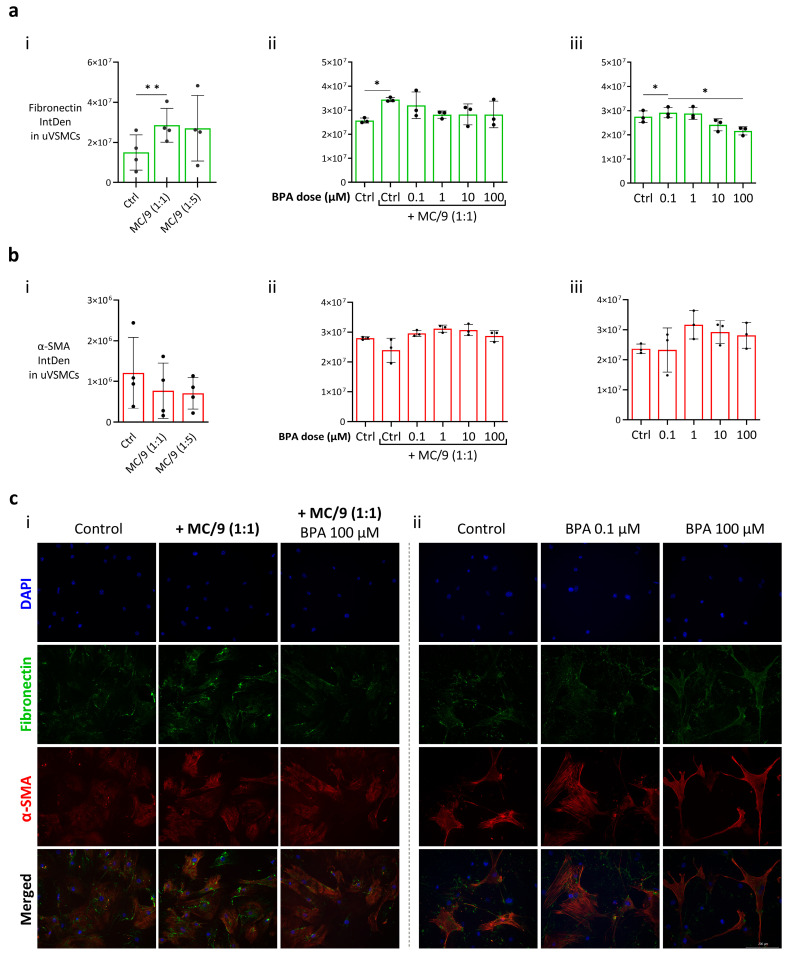
BPA exposure impairs MC/9 cells’ ability to significantly increase fibronectin expression in primary uVSMCs. The graphs show the IntDen value related to fibronectin (**a**) and α-SMA (**b**) fluorescent signals in primary uVSMCs after 24 h culture at different conditions: co-cultured with MCs at 1:1 or 1:5 cell-to-cell ratio ((**a**(**i**)); (**b**(**i**)); *n* = 3); exposed to BPA when cultured with MC/9 cells ((**a**(**ii**)); ((**b**(**ii**)); *n* = 3) or alone ((**a**(**iii**)); (**b**(**iii**)); *n* = 3). Repetitive measures ANOVA was conducted, and all groups’ means were compared to each other using Tukey’s multiple comparison post hoc test. Statistical significance was defined as *p* < 0.05. (**c**) Representative pictures depicting fibronectin (second row) and α-SMA (third row) fluorescent signals in uVSMCs after 24 h of BPA exposure, when cultured with MC/9 at a cell ratio of 1:1 (**i**) or alone (**ii**). *p* value: * ≤ 0.05; ** ≤ 0.001; BPA, bisphenol A; uVSMCs, uterine vascular smooth muscle cells; IntDen, integrated density; α-SMA, α-smooth actin; Ctrl, control (DMSO).

uVSMCs constitute the tunica media of SAs, where they exist in a quiescent contractile state, enabling the regulation of blood pressure and flow distribution [56]. Their transition to a synthetic phenotype at the onset of the remodeling enables their proliferation and migration out of the vessel, where they predominantly undergo apoptosis [40]. In the synthetic phenotype, uVSMCs exhibit a downregulation of the expression of contractile markers, including α-smooth actin (α-SMA) and calponin 1, and increased expression of extracellular matrix (ECM) proteins, such as fibronectin and collagen I [57]. To assess the impact of mouse MCs on uVSMCs phenotype switch promotion, we cultured primary uVSMCs together with MC/9 cells and determined the presence of the phenotype markers fibronectin and α-SMA. The results showed that the fibronectin fluorescence signal in uVSMCs was significantly increased after the co-culture with MC/9 cells (Figure 4a(i); *p* < 0.01), whereas there was no change related to the α-SMA signal (Figure 4b(i)). To test whether BPA was able to interfere with the observed impact of MCs on fibronectin expression, primary uVSMCs were cultured with or without MC/9 (cell-to-cell ratio 1:1) in BPA-containing medium. When uVSMCs were cultured with MC/9 cells, the presence of BPA was able to abrogate the significant increase in fibronectin signal observed in the untreated co-culture group (Figure 4a(ii)). On the contrary, when uVSMCs were exposed alone to BPA, we observed that the lowest dose tested (0.1 µM) was able to increase the fibronectin signal in (Figure 4a(iii)). However, no effect was identified at higher BPA concentrations. As regards α-SMA, BPA exposure did not affect the protein levels in uVSMCs in both experimental setups (Figure 4b(ii,iii)).

### 2.5. BPA Negatively Impacts on MC/9 Cells Promotion of uVSMCs Migration

**Figure 5:**
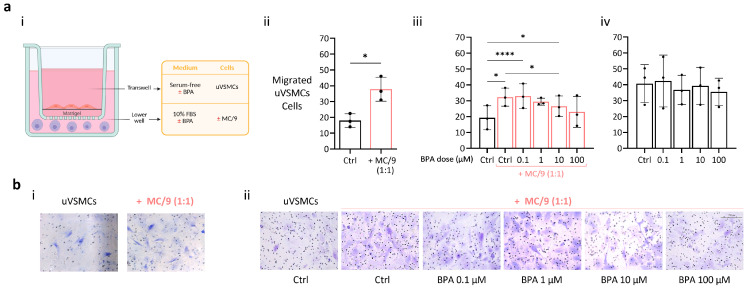
BPA exposure negatively impacts MC/9 cells’ promotion of primary uVSMCs migration, but it does not directly affect their ability to migrate. (**a**) Graphical scheme ((**i**); created in BioRender, accessed on 23 April 2025, https://BioRender.com/m81a623). The results show the number of migrating uVSMCs after 24 h in different experimental setups: in a coculture with MC/9 cells ((**ii**); 1:1 cell-to-cell ratio; *n* = 3); exposed to BPA at different concentrations, either cultured with MCs ((**iii**); *n* = 3) or alone ((**iv**); 1:1 cell-to-cell ratio; *n* = 3). Repetitive measures ANOVA was conducted, and all groups’ means were compared to each other using Tukey’s multiple comparison post hoc test. Statistical significance was defined as *p* < 0.05. *p*-value: * ≤ 0.05; **** ≤ 0.00001; (**b**) Representative images of crystal violet-stained uVSMCs cells that migrated to the lower side of the transwell membrane at different experimental conditions. BPA, bisphenol A; uVSMCs, uterine vascular smooth muscle cells; Ctrl, control (DMSO).

The enhanced migratory capacity of uVSMCs, driven by their phenotypic switch to a synthetic state, is a crucial factor for the proper progression of uSAR. For this reason, we first investigated whether MCs were able to influence mouse primary uVSMCs migration, as well as the consequences of BPA exposure, by performing a transwell migration assay. After 24 h, the cells co-cultured with MCs migrated significantly more compared to those cultured alone (*p* < 0.05) (Figure 5a(ii)). Then, to assess the impact of BPA on MCs’ ability to enhance the migration of uVSMCs, cells were left migrating in the presence of BPA-containing medium, with or without MC/9 cells. Interestingly, the exposure to 0.1 µM BPA did not impair the ability of MC/9 cells to promote uVSMC migration, as the number of migrated cells remained significantly higher compared to uVSMCs cultured alone. In contrast, at the concentration of 10 µM BPA, although migration was still significantly greater than uVSMCs alone, the number of migrated cells was significantly reduced compared to the untreated co-culture group (Figure 5a(iii)). Instead, no differences were detected in the migratory rates of uVSMCs cultured alone when exposed to BPA (Figure 5a(iv)). Representative images of the assay are shown in Figure 5b.

### 2.6. BPA-Induced Gene Expression Changes in SM9-2 and MC/9 Cells

**Figure 6:**
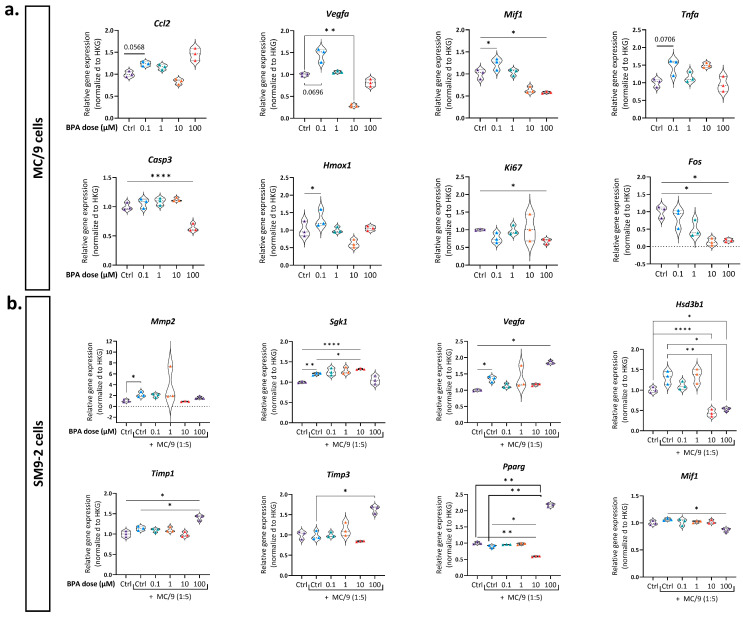
BPA impacts on the gene expression of MC/9 and SM9-2 cells following 24 h of co-culture. Violin plots show the results obtained from the qPCR analysis from MC/9 (**a**) and SM9-2 (**b**) cells after 24 h exposure to BPA, investigating the expression of genes related to invasion and vascular remodeling (*Mmp2*, *Sgk1*, *Timp1*, *Timp3*, *Vegfa*), inflammation and oxidative stress (*Ccl2*, *Tnfa*, *Mif1*, *Fos*, *Hmox1*), differentiation and hormones metabolism (*Pparg*, *Hsd3b1*), cell death and proliferation (*Casp3*, *Ki67*). Individual values represent the mean relative expression deriving from three technical replicates of single independent experiments (*n* = 3). Statistical analysis was performed using repeated-measures ANOVA, and all groups’ means were compared to each other using Tukey’s multiple comparison post hoc test. Statistical significance was defined as *p* < 0.05. *p* value: * ≤ 0.05; ** ≤ 0.001; **** ≤ 0.00001. HKG, housekeeping genes; BPA, Bisphenol A; *Ccl2*, C-C chemokine ligand 2; *Vegfa*, vascular endothelial growth factor; *Mif1*, macrophage inhibitory factor 1; *Tnfa*, tumor necrosis factor alpha; *Casp3*, caspase 3; *Hmox1*, heme oxygenase 1; *Ki67*, antigen kiel 67; *Fos*, c-Fos protein; *Mmp2*, matrix metalloproteinase-2; *Sgk1*, serum/glucocorticoid regulated kinase 1; *Hsd3b1*, hydroxy-delta-5-steroid dehydrogenase; *Timp1*, tissue inhibitor of metalloproteinases-1; *Timp3*, tissue inhibitor of metalloproteinase-3; *Pparg*; peroxisome proliferator activated receptor gamma; *Psmd4*, 26S proteasome non-ATPase regulatory subunit 4; *Tbp*, TATA box binding protein.

In order to understand how BPA exposure alters MC/9 cells’ functionality, ultimately having a negative impact on trophoblast invasion, we performed a gene expression analysis of both SM9-2 and MC/9 cells after 24 h of co-culture while exposed to BPA. We specifically focused on genes related to trophoblasts invasion and vascular remodeling (*Mmp2*, *Mmp9*, *Mif1*, *Sgk1*, *Timp1*, *Timp3*, *Vegfa*, *Pparg*), MCs activation and mediators (*Fos*, *Mcpt2*, *Tnfa*, *Tgfb1*, *Il6*, *Ccl2*, *Il13*, *Hdc*), cell death (*Casp3*, *Fas*, *Bcl2*), hormone receptors and metabolism (*Esr1*, *Esrra*, *Pgr*, *Hsd3b1*, *Cyp19a1*), proliferation (*Ki67*), and oxidative stress (*Hmox1*, *Sod1*). When SM9-2 cells were co-cultured with MC/9 cells, the expression of the invasion and remodeling-related genes *Mmp2* (matrix metalloproteinase 2), *Sgk1*, and *Vegfa* significantly increased, suggesting a potential mechanism by which MCs enhance trophoblast invasiveness. Interestingly, BPA exposure did not downregulate the expression of these genes; however, at 100 µM BPA, we observed a significant upregulation of the *Timp1* and *Timp3* genes, which encode matrix metalloproteinase inhibitors, alongside a marked downregulation of the *Mif1* gene, involved in the regulation of trophoblasts’ invasion [58]. Additionally, the oxidative stress marker *Hmox1* was significantly upregulated, while *Hsd3b1*, essential for progesterone synthesis, was reduced at both 10 and 100 µM BPA. Regarding MC/9 cells, exposure to 0.1 µM BPA led to a significant increase in *Mcpt2*, *Ccl2*, *Tnfa*, *Vegfa*, *Hmox1*, and *Mif1* expression, indicative of a pro-inflammatory activation of MCs. Moreover, at 100 µM BPA, the expression of *Casp3* and *Ki67* was markedly reduced, while *Bcl-2* increased. On the other hand, the exposure to 10 and 100 µM BPA resulted in a significant decrease in *Fos* expression, a gene known to be involved in MCs activation. No significant differences were observed in the expression of genes related to hormone receptors or of other immune mediators, while the expression of *Pgr*, *Cyp19a*, and *Mmp9* genes could not be detected in any of the cell lines (Figure 6 and Figure S2).

## 3. Discussion

The role of MCs in uSAR has long remained unexplored. In our initial study, aimed at filling this knowledge gap, we used a mouse model characterized by MCs deficiency (*Kit^W-sh/W-sh^*). MC-deficient pregnant mice exhibited signs of impaired uSAR at gd10, having spiral arteries with significantly smaller lumens and a higher wall-to-lumen ratio. These dams were also characterized by a lower number of implantations and a higher percentage of fetal death. This severe pathological phenotype was recovered by the infusion of bone marrow-derived mast cells (BMMCs), confirming that the absence of MCs is enough to cause insufficient uSAR and related pregnancy complications [46]. However, the mechanisms behind MCs’ role in uSAR are still under study. Our recent work suggests that MCs likely act through their mediators by positively influencing the cellular processes leading to a successful vascular remodeling process. More precisely, they improve the functionality of cells involved in the remodeling. We indeed showed that human MCs (HMC-1 cells) were able to promote extravillous trophoblasts (EVTs, HTR-8 cells) proliferation and invasion, as well as the proliferation, migration, and phenotype switch of primary human uVSMCs [47]. In the present study, we found that similar results are also observed when using cell lines and primary cells derived from mice: MC/9 cells are able to increase SM9-2 trophoblast invasion at both 1:1 and 1:5 cell-to-cell ratios, with the latter leading to a more significant effect.

Similarly, MC/9 cells enhance the migration and fibronectin expression of mouse primary uVSMCs. This suggests that MCs may contribute to the transition of uVSMCs from a contractile to a synthetic phenotype, characterized by increased synthesis and secretion of extracellular matrix proteins, such as fibronectin and collagen I, along with enhanced proliferative and migratory features. The composition of the ECM is critical for uVSMCs phenotype modulation, and the presence of fibronectin further supports the switch to the synthetic state [59]. This phenotypic change enables their migration away from the vessel, leading to a marked reduction in arterial resistance and pulsatility, ultimately resulting in increased blood flow to the uterus at a lower velocity [60]. While MCs are not the sole drivers of uVSMC phenotypic transition, we believe they might work in conjunction with uNKs, the best-known regulators of this process. This hypothesis is supported by our previous study, which demonstrated that in mice deficient in either MCs or uNKs, one cell type can compensate for the absence of the other in regulating uSAR, suggesting their redundant functions in this important vascular adaptation [45]. Interestingly, it has been shown that uNKs are able to initiate the phenotype change in uVSMCs even before the invasion of trophoblasts, through the release of angiogenic growth factors, like angiopoietin 2 (Ang 2) [61]. Therefore, in future studies, it would be interesting to further explore the shared functions and interactions of MCs and uNKs in the context of uSAR and placenta development, focusing also on the timing of their involvement.

In contrast to our previous findings with human cell lines, mouse MCs did not enhance the proliferation of SM9-2 trophoblasts. These results are not surprising, since even though the invasion routes of human and mouse trophoblasts are comparable (interstitial and endovascular), the differentiation pathways and the phenotypic profiles of these cells are very different [62]. Consequently, the interaction between MCs and invasive trophoblasts could exhibit differences between the species.

Acknowledging that MCs’ presence and activity are crucial for the successful outcome of uSAR, we then sought to test whether the exposure to BPA—a ubiquitously prevalent environmental chemical posing a considerable threat to women’s reproductive health—could interfere with their essential functions. Numerous studies conducted on mouse models have already shown that BPA exposure can lead to reduced female fertility, implantation problems, altered ovarian functions, and hormonal imbalance [26,27,63,64]. 

Concerning the impact of BPA on MCs, previous studies reported that exposure to this chemical could exacerbate MC activation, leading to an increased release of histamine and inflammatory cytokines, such as IFN-γ, TNF-α, and IL-6 [49,50,51]. However, the consequences of these alterations on physiological processes dependent on MC activity, such as uSAR, as well as on other cell types regulated by MC-derived signals, remain unclear. To address this gap, we started by assessing the presence of at least one BPA binding site in the cell lines and primary cells used in our study. We showed that they express ERα, confirming that they can be potential targets of BPA. However, it is highly probable that their receptor repertoire includes additional sites for BPA interaction. Indeed, although BPA activity is prevalently described as estrogenic, its mode of action is far more complex. It involves not only nuclear and membrane-bound estrogen receptors, but also androgen, glucocorticoid, thyroid hormone, and PPARγ receptors [65].

To address our research question, we repeated the previously described experiments, this time exposing the cells to different concentrations of BPA. Since trophoblasts, uVSMCs, and MCs are all potential targets of BPA, we used two setups for each experiment: (1) SM9-2 cells or uVSMCs co-cultured with MC/9 cells and (2) SM9-2 cells or uVSMCs cultured alone. This approach enabled us to discern whether BPA has a direct influence on the functionality of SM9-2 cells or uVSMCs, and/or if its effects are indirect and caused by the interference with MC/9 cell activity.

The murine trophoblast SM9-2 cell line was chosen as a model in our study as these cells are characterized by a highly invasive phenotype [66,67]. For this reason, they are suitable to represent the mouse trophoblast populations involved in the invasion process and in uSAR, more precisely, spiral artery trophoblast giant cells (SA-TGCs) and glycogen trophoblast cells [62]. The MC/9 cell line was instead selected as it is the best characterized murine MC cell line in the literature. Notably, these cells express the MC key markers and receptors FcεRI and c-Kit, which are central to their activation, survival, and function [68]. Moreover, although MC/9 cells are mainly used as a model for mucosal MCs, they also have phenotypic traits of connective tissue MCs, as they express the Mcpt5 chymase [68,69]. In our previous work, we demonstrated that Mcpt5 contributes to MCs’ role in uSAR. Indeed, the addition of this chymase in the culture medium was able to enhance the invasion of human trophoblasts, producing effects comparable to those observed in the presence of MCs themselves [47].

The choice of the BPA concentrations used was made taking into account human exposure levels, established toxicological guidelines, and BPA accumulation, distribution, and metabolism within the body. The concentration of BPA within biological human samples can vary highly based on plenty of factors, such as age, sex, and geographical location [70]. With regard to pregnant women, circulating BPA levels range from 0.3 to 18.9 ng/mL, while the highest concentration found in urine is 31.9 μg/L (approximately 0.1396 µM) [71,72,73]. It is important to mention that BPA concentrations are usually reported as free BPA (unconjugated, active form), conjugated BPA (metabolized inactive form, present either as BPA-glucuronide or BPA-sulfate), or total BPA (combination of both forms) [74]. The range of unconjugated BPA—the lipophilic, bioactive form responsible for endocrine disruption– has been reported in placenta tissues at levels ranging from 1 up to 165 ng/g [75]. The concentration of BPA in the placenta-fetal unit can be higher than that found in maternal serum [10]. This discrepancy is largely attributed to the presence of β-glucuronidase in the placenta, which deconjugates BPA-glucuronide back to its active parent compound. Moreover, the fetal liver is considerably less efficient at metabolizing BPA due to its lower expression of conjugative enzymes, such as UDP-glucuronosyltransferases (UGTs) and sulfotransferases (SULTs), resulting in the increased persistence of bioactive BPA in the fetal compartment [76,77]. Since our study is based on in vitro assays, we also considered the “in vitro—in vivo scaling factor”, a concept widely used in toxicology. As defined by Albrecht in a letter to the editor published in 2020, this factor represents the extent “by which in vitro concentrations have to be higher than the corresponding in vivo plasma concentration in order to cause similar biological effects in the target cells”. Accordingly, this article suggests testing a range of concentrations up to 200-fold higher than blood levels [78,79]. Taking these points into consideration, we decided to test the following range of concentrations to look into several BPA exposure scenarios: 0.1, 1, 10, 100 μM.

Having performed an invasion assay, we found that the presence of BPA in the culture medium, at any of the tested concentrations, impaired the ability of MC/9 cells to significantly foster SM9-2 trophoblast invasion. Notably, BPA was not able to affect SM9-2 invasion rates when these cells were cultured without MC/9 cells. This finding suggests that BPA indirectly impacts trophoblast invasion by disrupting the function of MCs. Our results do not align with most of the existing data on the direct impact of BPA on trophoblast invasion. Nonetheless, comparisons are challenging due to variations in the cell lines used and the different exposure conditions, which often involve the pre-exposure of the cells to BPA before the assay [80]. In the study by Zi-Yi Wang et al., BPA at concentrations of 0.01, 1, and 100 μM significantly reduced the invasion rates of human BeWo trophoblasts in a dose-dependent manner. However, in this case, the invasion and exposure time was 48 h, and the trophoblasts were co-cultured with endometrial cells plated in the lower well. Additionally, different percentages of FBS were used in the medium compared to those used in our assay [81]. In a later investigation, Wei P et al. explored the impact of BPA pre-exposure (48 h) on the invasion of human HTR-8/SVneo trophoblasts. Their results showed that pre-treating the cells with BPA at concentrations of 10^−8^, 10^−7^, and 10^−6^ M significantly reduced their invasion ability [82]. On the other hand, Spagnoletti et al. obtained results consistent with ours in an invasion assay using HTR-8/SVneo cells pre-exposed to BPA for 24 h at concentrations of 10^−15^, 10^−13^, 10^−11^, 10^−9^, and 10^−7^ M [83]. Still, all these findings relate to human trophoblasts, making our study the first to investigate the impact of BPA on the invasion properties of mouse trophoblasts in vitro.

With respect to the proliferation assay, we only tested the direct impact of BPA on SM9-2 trophoblasts, as MC/9 cells did not improve their proliferative behavior. Hence, we exposed the cells to the chemical for up to 72 h to assess potential changes in their proliferation and viability. We observed that only the 100 µM concentration was able to significantly decrease both proliferation (at 24, 48, and 72 h) and cell viability (at 48 and 72 h). Existing data on BPA’s effects on trophoblast proliferation remain inconsistent. As highlighted in a review by Adu-Gyamfi et al., these discrepancies likely arise from variations in trophoblast cell lines and methods used [84]. Nonetheless, most studies report that BPA is able to decrease human trophoblast proliferation at both low and high doses (0.1 to 100 nM and 100 µM) following 24 and 48 h of exposure [85,86,87].

Next, we observed that BPA exposure interfered with the ability of MCs to significantly increase fibronectin expression in uVSMCs (at all doses) and enhance their migration (at 1 and 100 µM). However, BPA had no significant effect on these parameters when uVSMCs were cultured alone, except at the lowest concentration (0.1 µM), which increased fibronectin expression after 24 h of exposure. These findings are particularly interesting because, while no specific data are available on the effects of estrogen exposure on uVSMCs functionality, estrogen has been shown to induce fibronectin expression and promote ECM assembly in human breast cancer cells [88]. For this reason, we can only speculate that at this low dose, BPA may exhibit estrogen-mimic activity, though this should be confirmed by further experiments.

To elucidate the mechanisms by which MC/9 cells support trophoblast functionality, particularly their invasive ability, and to assess the impact of BPA, we analyzed the expression of relevant genes in both cell types after 24 h of co-culture and BPA exposure. We found that when co-cultured with MC/9 cells, SM9-2 trophoblasts exhibited significantly increased expression of *Mmp2*, *Sgk1*, and *Vegfa* genes. These findings support the observed pro-invasive influence of MC/9 cells on trophoblasts and align with our hypothesis that MCs contribute to uSAR, as these genes are key regulators of matrix remodeling, invasion, and angiogenesis [89,90,91,92]. When examining the gene expression changes in MC/9 cells upon BPA exposure, we found that the lowest BPA dose (0.1 µM) triggered the increased expression of *Vegfa*, *Mif1*, and *Mcpt2*, indicative of MCs activation, as well as of *Tnfa*, *Ccl2*, and *Hmox1* genes, showing a pro-inflammatory tendency [93,94,95,96]. Of note, the highest concentration of BPA led to a significant decrease in Mif1, which encodes a multifunctional cytokine highly present at the fetal–maternal interface that supports trophoblasts differentiation and invasion [58]. Furthermore, at higher concentrations (10 and 100 µM), BPA exposure led to a reduction in *Fos* expression. Given that c-Fos is a transcription factor typically expressed upon activation and degranulation, its downregulation may indicate a dampened activity of MC/9 cells [97]. Exposure to the highest BPA dose (100 µM) also resulted in a marked decrease in *Ki67*, indicating reduced proliferation of MCs. This reduction is likely due to cell cycle arrest, as evidenced by the concurrent significant decrease in Caspase-3 (*Casp3*) expression [98].

Regarding SM9-2 cells, while BPA exposure did not specifically downregulate the expression of the pro-invasive genes, which increased under MC/9 cells’ influence, it triggered other changes that could have impacted their functionality. Notably, exposure to the highest BPA concentration (100 µM) increased the expression of *Timp1* and *Timp3*, which encode inhibitors of matrix metalloproteinases (Timps). These changes indicate that high-dose BPA disrupts the balance between metalloproteinases (Mmps) and their inhibitors—a balance that is essential for ECM degradation and, consequently, for the invasion of trophoblasts during uSAR [99]. These findings are consistent with previous research indicating that BPA exposure alters Mmps and Timps protein levels, leading to reduced invasion capability in human BeWo trophoblasts [26]. Furthermore, the 100 µM dose led to a significant decrease in *Mif1* gene expression, which also has an autocrine effect in the stimulation of trophoblast own invasion [58]. At concentrations of 10 and 100 µM, BPA exposure led to a decreased expression of the *Hsd3b1* gene in SM9-2 cells, responsible for the synthesis of the 3β-Hsd1 enzyme that converts pregnenolone into progesterone—an essential process for trophoblast differentiation and pregnancy maintenance [55,100]. *Pparg* gene expression was also significantly altered in trophoblasts exposed to 10 and 100 µM BPA. Interestingly, this gene—abundantly expressed in all mouse placenta zones—is involved in the maintenance of the undifferentiated state of trophoblasts, and in the regulation of syncytiotrophoblasts differentiation in the labyrinth zone, the area of the mouse placenta where the nutrients are exchanged [101,102].

Overall, we believe that the gene expression changes observed in the trophoblasts are also a consequence of altered MC/9 cells functionality. Indeed, our assays did not reveal any direct effect of BPA on SM9-2 cell invasion. Rather, the negative impact appears to stem from BPA’s disruption of MC/9 cell functionality, reducing their ability to foster trophoblast invasion. It is clear that when exposed to the highest BPA dose (100 µM), the observed reduction in MCs proliferation and activity (*Ki67*, *Casp3*, *Mif1*, *Fos*) seem to create an anti-invasive microenvironment, reflected in SM9-2 cells with the significant increased expression of *Timp1* and *Timp3* genes, and altered expression of genes involved in their differentiation (*Hsd3b1* and *Pparg*). Although we showed that even the lowest dose of BPA was able to activate MC/9 cells and trigger a pro-inflammatory behavior, the underlying mechanisms of low doses remain elusive. However, it is important to note that BPA can also act via non-genomic pathways that modulate cellular responses without altering gene transcription—thus, its effects at lower doses might not be fully captured by transcriptomic analyses [103]. In fact, though BPA can target classic cytoplasmatic and nuclear ERs, it can also bind to membrane ERs, activating downstream pathways—including the mitogen-activated protein kinase (MAPK) one—which ultimately regulate cell proliferation, apoptosis, and differentiation [14]. In MCs, it was observed that estrogen could induce their degranulation in vitro through GPER, a membrane ER that triggers non-genomic rapid signaling responses resulting in calcium mobilization, cAMP production, and kinase phosphorylation [52,104]. Degranulation is MC primary response to external stimuli, a process involving the release of pre-formed mediators such as histamine, proteases, and cytokines. Interestingly, BPA-mediated activation of GPER can trigger the activation of the MAPK pathway and alter the balance between cell proliferation and apoptosis, a mechanism that was also correlated to BPA’s role in tumorigenesis and cancer progression [105,106,107]. For this reason, we can speculate that BPA targeting of GPER in MCs might be a possible mechanism through which this chemical interferes with the normal regulation of their activation and degranulation, ultimately affecting their role in vascular remodeling.

Accordingly, additional investigations are required to further validate our hypothesis that BPA interferes with MC/9 activity, ultimately impairing the critical release of mediators and the creation of a supportive microenvironment necessary for successful trophoblast invasion and proper uVSMCs functions.

As failure of uSAR is strongly associated with the onset of preeclampsia—a hypertensive disorder whose underlying causes are only partially understood—a confirmation of our hypothesis could provide important insights not only into the reproductive hazards posed by BPA, but also into pathophysiological mechanisms underlying this pregnancy complication [36,108]. Such knowledge could build more awareness of environmental risk factors and contribute to the prevention of this serious disorder.

## 4. Materials and Methods

### 4.1. Primary Cells and Cell Lines

Mouse primary uVSMCs (#C57-6213, Cell biologics, Chicago, IL, USA), were grown on flasks or 18 × 18 mm cover slides (Th. Geyer, Renningen, Germany) coated with gelatin (Cell biologics, USA) in complete smooth muscle cell medium (#M2268, Cell biologics, USA) containing 10% fetal bovine serum (FBS, Pan-Biotech, Aidenbach, Germany), 0.1% insulin, 0.1% epidermal growth factor, 0.1% fibroblast growth factor and 1% L-Glutamine. The mouse extravillous trophoblast cell line SM9-2 (kindly provided by Joan Hunt and David Wheaton, University of Kansas Medical Center, Kansas City, MO, USA) was cultured in RPMI 1640 medium (Gibco, Darmstadt, Germany) supplemented with 20% FBS, 1% penicillin/streptomycin (P/S, Thermo Fisher Scientific, Waltham, MA, USA), 1 mmol/L sodium-pyruvate (Sigma-Aldrich, St. Louis, MO, USA) and 0.05 mM β-Mercaptoethanol (Sigma-Aldrich, USA). The mouse mast cell line MC/9 (#CRL-2036, ATCC, Jena, Germany) was maintained in RPMI 1640 medium supplemented with 10% FBS, 1% P/S, 0.1 mM MEM non-essential amino acids solution (Gibco, Germany), 0.05 mM β-Mercaptoethanol, and 30 ng/mL of recombinant mouse IL-3 (Sigma-Aldrich, USA). All cells were cultured in T25 or T75 flasks at 37 °C with 5% CO_2_ in a humidified atmosphere.

### 4.2. ERα Immunostaining

SM9-2, uVSMCs, and MC/9 cell suspensions were used to prepare cell smears on 25 × 75 mm glass slides (Superfrost plus adhesion microscope slides, Epredia Holdings Ltd., Portsmouth, NH, USA), which were left to dry overnight. Cells were fixed for 8 min in frozen acetone (−20 °C, Sigma-Aldrich, USA) and left to dry at room temperature (RT). Circular areas around the fixed cells were drawn using an Elite PAP pen (Diagnostic Biosystem, Pleasanton, CA, USA). Following, cells were washed (3 times, 5 min) in 1× tris-buffer saline (TBS) (Tris, Carl Roth, Germany), permeabilized for 10 min in 0.1% triton X-100 (Sigma-Aldrich, USA), and blocked in a 30 min incubation step with 3% bovine serum albumin (BSA, Sigma-Aldrich, USA) in a humidity chamber. At this point, an overnight staining was performed using an Estrogen Receptor Alpha monoclonal antibody (clone 33, Thermo Fisher Scientific, USA) at a concentration of 10 µg/mL in the humidity chamber at 4 °C. The following day, a washing step was carried out (3 times, 5 min in 1× TBS), and subsequently cells were incubated for 2 h at RT with AF555 goat anti-mouse IgG (Thermo Fisher Scientific, USA) diluted 1:500. After removing the secondary antibody in a washing step (3 times, 5 min in 1× TBS) cells were mounted using VECTASHIELD^®^ Antifade Mounting Medium containing DAPI (Vector laboratories, Stuttgart, Germany). Slides were analyzed at Keyence microscope BZ-X810 (Keyence Corporation, Osaka, Japan), and pictures were taken using a magnification of 20× and 40×.

### 4.3. Transwell Invasion Assay

SM9-2 cells or uVSMCs were starved in serum-free medium for 16 h in a T75 flask. The assay was conducted using 8 µm pore size transwell inserts (Corning, Steuben County, NY, USA) in 24-well culture plates. Transwells were pre-coated using 0.5 mg/mL growth factor-reduced Matrigel (Corning, USA), and afterwards 1 × 10^5^ SM9-2 cells or 5 × 10^4^ uVSMCs were seeded in serum-free medium on top of the matrigel layer. In the lower well, 10% FBS medium was added with or without MC/9 cells (cell-to-cell ratios 1:1 and 1:5). In the BPA exposure experiments, the medium supplied contained BPA at concentrations of 0.1, 1, 10, and 100 µM. BPA-containing medium was prepared starting from a stock solution of BPA (Sigma-Aldrich, USA) dissolved in DMSO (dimethylsulfoxide; Sigma-Aldrich, USA) at a concentration of 22.829 mg/mL (0.1 M). In the control groups of every experiment performed, DMSO (1:1000 dilution) was added to the refreshed medium to match the highest DMSO concentration present in the BPA-containing wells. Cells were incubated in the incubator (37°, 5% CO_2_) for 24 h. Next, non-invasive cells on the upper side of the transwell membrane were removed with cotton swabs (Süsse Labortechnik, Gudensberg, Germany) while the remaining cells were fixed using 70% ethanol (Bioethanol, Th. Geyer, Germany) and stained with 0.2% crystal violet (Sigma-Aldrich, USA). Five pictures of each membrane were taken using a Keyence microscope at a 20× magnification, and the invasive cells were then counted using ImageJ software v1.54f (U.S. National Institutes of Health, Bethesda, MD, USA). The number of invasive cells was calculated as the mean of the cells counted in the 5 pictures.

### 4.4. Proliferation and Viability Assay

Cell TraceTM CFSE Cell Proliferation Kit (Thermo Fisher Scientific, USA) was used to assess SM9-2 cells’ proliferation when cocultured with MC/9 cells or exposed to BPA for 24, 48, and 72 h. A total of 2 × 10^6^ cells was washed with 1× Dulbecco’s Phosphate-Buffered Saline (PBS, BioWest, Düsseldorf, Germany) and then incubated with 5 µM CFSE solution (20 min, 37 °C, in the dark) prepared according to the manufacturer’s instructions. An amount of cell culture medium equal to 5 times the volume of the CFSE solution used was then gently added to the cell suspension to stop the staining process. After 5 min of resting time (RT, shielded from light), the cells were centrifuged (8 min, RT, 1200 rpm). The supernatant containing the staining solution was then aspirated, and the cells were resuspended in warm medium. Following a 10 min incubation step at 37 °C, cells were counted and plated (9 × 10^3^/well) in three 12-well plates (one for each time point), containing 2.5 mL of medium/well. For the coculture experiments, after 16 h in the incubator (37 °C, 5% CO_2_), the medium was changed with fresh medium containing 30 ng/mL of recombinant mouse IL-3. Transwells were added and filled with 1.5 mL of IL-3-supplemented medium, and MC/9 cells at a concentration of 1:1 or 1:5 cell-to-cell ratios were plated within them. For the BPA exposure experiments, the old medium was instead replaced by BPA-containing medium at different concentrations (0.1, 1, 10, and 100 µM), and no MC/9 cells were added. After 24, 48, and 72 h, SM9-2 cells were washed and collected from the plate. To assess viability, cells were stained with eBioscience™ Fixable Viability Dye eFluor™ 506 (FVD, Thermo Fisher Scientific, USA) at a concentration of 1:500 (30 min, 4 °C, protected from light). Cells were then centrifuged and resuspended in PBS containing 1% FBS, to be then examined at the Attune NxT Flow Cytometer (Thermo Fisher Scientific, USA). Flow cytometry data were analyzed with FCS Express 7 Research Edition (De Novo Software, Pasadena, CA, USA). The median fluorescence intensity (MFI) related to the CFSE dye was used to assess proliferation, with decreasing signal intensity indicating increased cell division (Figure S1).

### 4.5. Immunofluorescence

2 × 10^4^ mouse primary uVSMCs were seeded on 18 × 18 mm cover slides (Th. Geyer, Germany) placed in 6-well cell culture plates (Corning, USA) containing 2.5 mL medium/well, and let grow for 16 h. Afterwards, MC/9 cells (cell-to-cell ratio 1:1 and 1:5) were added to 0.4 µm pore size transwell inserts for 6-well plates (Corning, USA) filled with 1.5 mL medium. Control wells did not contain MC/9 cells, and 4 mL of fresh medium was replaced instead. For the BPA exposure experiments, uVSMCs were cultured in BPA-containing medium at concentrations of 0.1, 1, 10, and 100 µM. In the coculture experiments, MC/9 cells were added to transwells containing BPA-containing medium at the same concentrations. Cells in the control groups were plated in medium containing DMSO (1:1000 dilution). After 24 h, the uVSMCs on the cover slides were rinsed with 1x PBS and fixed with 4% paraformaldehyde (Carl Roth, Karlsruhe, Germany) for 10 min. Cells were then permeabilized with 0.5% Triton X-100 for 3 min and blocked in 1% BSA for 30 min. The cover slides were incubated with the primary antibody mixture of anti-human fibronectin (1:100, ab2413, Abcam, Waltham, MA, USA) and mouse anti-human α-SMA (1:50, clone 1A4, #M0851, Dako, Agilent Technologies, Santa Clara, CA, USA) for 2 h at RT. After removing the antibody mix in a washing step (3 times, 5 min, with 1× PBS), the slides were incubated with the secondary antibody mixture of goat anti-mouse AF555 (1:500, A21424, Invitrogen, Gräfelfing, Germany), anti-rabbit-AF488 (1:1000, #ab150077, Abcam, USA) for 1 h at RT. After washing (3 times, 5 min, with 1× PBS), the cover slides were transferred to 25 × 75 mm glass slides and mounted with VECTASHIELD^®^ Antifade Mounting Medium with DAPI. Three images of each well were captured using a Keyence microscope at a 20× magnification. The fluorescent signal detected was measured as integrated density (IntDen) with Image J software v1.54f, and used as an indicator of the degree of protein expression within the cells.

### 4.6. Gene Expression Analysis

SM9-2 cells (7 × 10^4^) were seeded in 2.5 mL of complete medium in 6-well plates designed for transwell insertion (Corning, USA). Approximately 16 h later, the medium was replaced with fresh medium containing 30 ng/mL of recombinant mouse IL-3, and either DMSO (control groups, 1:1000) or BPA (0.1, 1, 10, 100 µM). MC/9 cells were then cocultured (cell-to-cell ratio 1:5) with the trophoblasts using 0.4 µm pore size transwell inserts, each filled with 1.5 mL of IL-3-supplemented medium containing either DMSO or BPA. After 24 h, SM9-2 and MC/9 cells were harvested, and their pellets were resuspended in 300 µL of TRIzol reagent (Thermo Fisher Scientific, USA), and the total RNA was isolated following the manufacturer’s instructions. RNA purity and concentration were measured using the NanoDrop system (Thermo Fisher Scientific, USA). cDNA was synthesized with the ImProm-IITM Reverse Transcription System (Promega, Walldorf, Germany) using oligo(dT)18 and random hexamer primers (Thermo Fisher Scientific, USA). Primers (Table S1) for semi-quantitative PCR (qPCR) were designed using the web-based Primer3Plus package (www.primer3plus.com, accessed on 11 November 2024). The qPCR was performed either on the Biomark HD system (StandardBioTools, San Francisco, CA, USA) with BioMark™ Dynamic Array Integrated Fluidic Circuits using EvaGreen^®^ Dye (Biotium, Fremont, CA, USA) as DNA binding dye, or on the LightCycler 480 (Roche, Ludwigsburg, Germany) according to the manufacturer’s recommendations. For both platform protocols, the BIOTAQ DNA polymerase (BioCat, Heidelberg, Germany) was used. The stability of the reference genes was checked with the geNorm algorithm implemented in the open-source qbase+ data-analysis software (www.qbaseplus.com). To quantify the relative expression, the expression of the genes of interest was normalized to the geometric mean expression of reference genes TATA-box binding protein (*Tpb*) and proteasome 26S subunit (*Psmd4*), used to calculate the 2^−ΔΔCt^ value [109].

### 4.7. Statistical Analysis

Statistical data analysis was performed using GraphPad Prism software version 8.0 (GraphPad, Boston, MA, USA). Statistical significance was defined as *p* < 0.05. Data were tested for normal distribution using the Shapiro–Wilk test. The statistical analysis was performed using repeated-measures ANOVA, except for the proliferation assay data, which were analyzed with two-way ANOVA. The means of all groups were compared to each other using Tukey’s multiple comparison post hoc test. When data were not normally distributed, Friedman’s test was performed, followed by Dunn’s multiple comparisons post hoc test. All assays consisted of at least three independent experiments, with each experimental group tested in duplicate.

## 5. Conclusions

Our in vitro study confirmed that murine MCs can promote the invasion of SM9-2 trophoblasts, as well as the synthesis of fibronectin and the migration of mouse primary uVSMCs. These findings align with our human cell line study and further validate the contribution of MCs to uSAR-related processes. Additionally, we showed that MC/9 cells enhance the expression of invasion and angiogenesis-related genes in SM9-2 cells, shedding light on the mechanisms underlying MC-driven invasion promotion.

**Figure 7:**
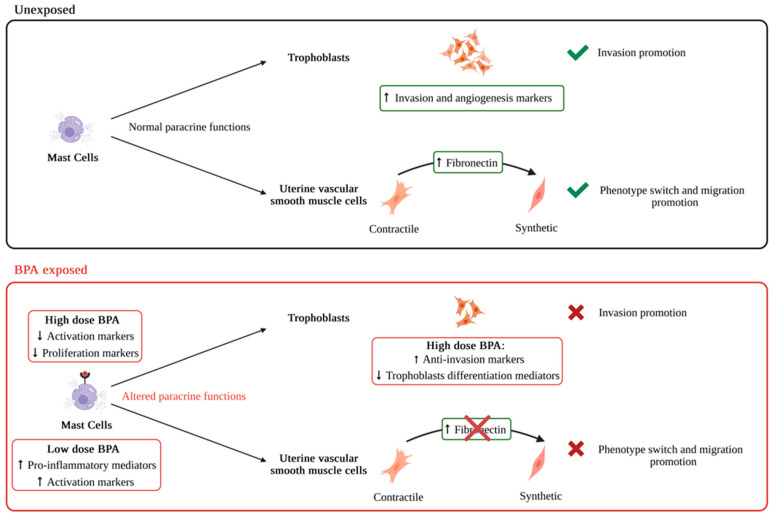
BPA impairs MC-mediated promotion of cellular processes associated with uSAR. Graphical summary of the key findings reported in the present study, resulting from functional in vitro assays performed with murine cell lines (MCs and trophoblasts) and primary cells (uVSMCs). Created in BioRender, accessed on 12 September 2025, https://BioRender.com/k5rg7dg. BPA, Bisphenol A; uSAR, uterine spiral artery remodeling; MCs, mast cells; uVSMCs, uterine vascular smooth muscle cells.

BPA exposure, across a range of concentrations (0.1 to 100 µM), compromised the ability of MC/9 cells to support these key cellular functions in both SM9-2 cells and uVSMCs—functions that are essential for proper uSAR (Figure 7). At higher BPA concentrations (10 and 100 µM), this disruption was likely driven by the downregulation of genes involved in MCs proliferation and activation. This had an indirect effect on the expression of invasion and differentiation-related genes in SM9-2 cells. On the other hand, the effects of BPA at low concentrations may act through non-genomic pathways, potentially interfering with the degranulation of MCs and their supportive role in trophoblast invasion. Collectively, these mechanisms could underlie the broader negative impact of BPA on trophoblast and uVSMC functions, ultimately impairing the uSAR process. Further research is needed to validate this hypothesis, particularly by examining BPA’s influence on MCs’ degranulation and its modulation of rapid, non-genomic signaling pathways in both MCs and invasive trophoblasts.

## Data Availability

The raw data supporting the conclusions of this article will be made available by the authors on request.

## Supplementary Materials

The supporting information can be downloaded at: https://www.mdpi.com/article/10.3390/ijms26199706/s1.
