# Supplementary material for: Bisphenol A Interferes with Mast Cell-Mediated Promotion of Cellular Processes Critical for Spiral Artery Remodeling
Source: Int J Mol Sci. 2025 Oct 5;26(19):9706. doi: 10.3390/ijms26199706 (PMC12525175; doi:10.3390/ijms26199706)
Supplement: Supplementary file 1 [file ijms-26-09706-s001.zip › ijms-3795774-supplementary.pdf]

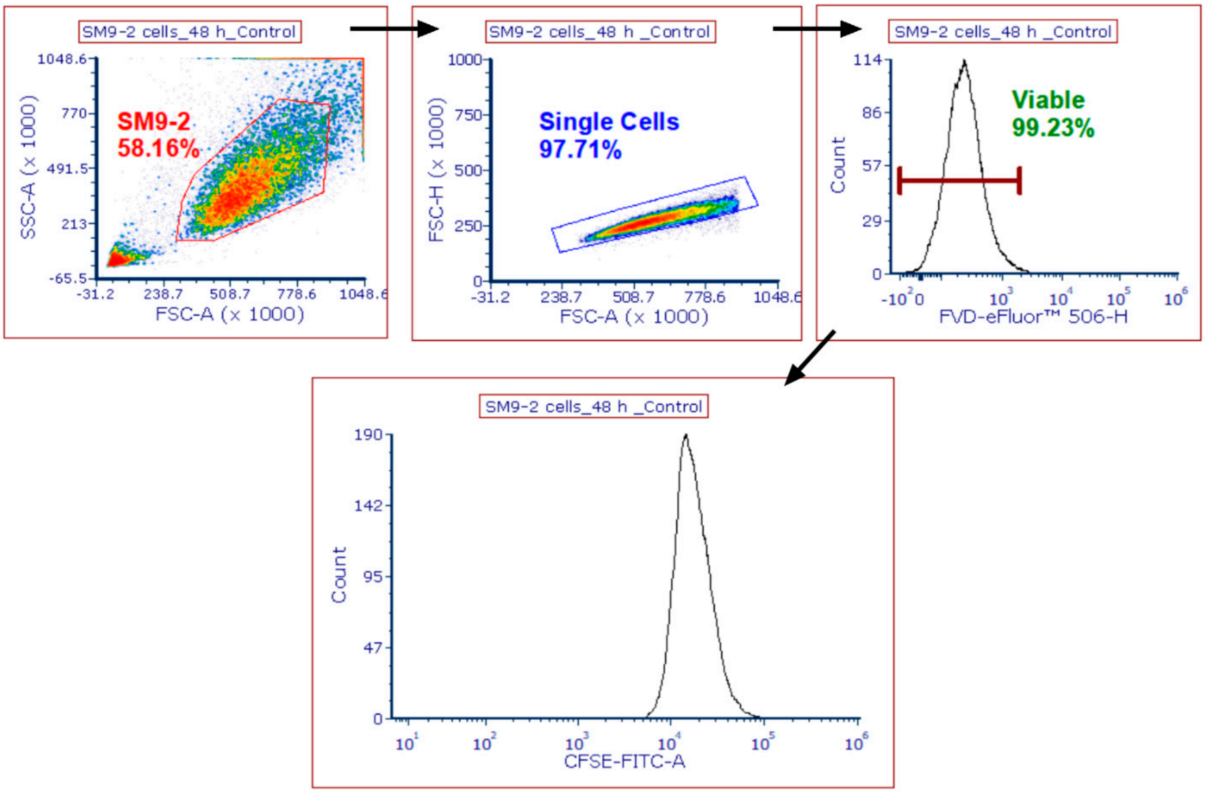

Figure S1. Gating strategy related to CFSE-based proliferation assay.

Table S1. List of the primers used for the qPCR.

Gene	Forward primer	Reverse primer
Bcl-2	GGTGAACTGGGGGAGGATTG	GCATGCTGGGGCCATATAGT
Casp-3	GAGGCTGACTTCCTGTATGCTT	AACCACGACCCGTCCTTT
Ccl2	GGCTGGAGAGCTACAAGAGG	TCTTGAGCTTGGTGACAAAAAC
Fos	ACAGATCTGCGCAAAAGTCC	CAGCCTTTCCTACTACCATTCC
Esr1	TTACGAAGTGGGCATGATGA	CCTGAAGCACCCATTTCATT
Essra	AGGTGGACCCTTTGCCTTTC	CAGGTTCAACCACCAGCAGA
Fas	GGAGACAGGATGACCCTGAA	AGCAGCTGGACTTTCTGCTC
Hdc	TGGTGCCTGTGTTTGTCTGT	TACTCGATGCCCTCCAGGAA
Hmox1	AGGCTAAGACCGCCTTCCT	TGTGTTCCTCTGTCAGCATCA
Hsd3b1	TGCAGACAAAGACCAAGGTG	CTTGAACACAGGCCTCCAAT
Il13	CGGTGCCAAGATCTGTGTCT	GTTGGTCAGGGAATCCAGGG
Il6	TACCACTTCACAAGTCGGAGGC	CTGCAAGTGCATCATCGTTGTTC
Ki67	GCTGTCCTCAAGACAATCATCA	GGCGTTATCCCAGGAGACT
Mcpt2	CCAGGACAGCAACACCCTAG	TAGGCCATGTAAGGACGGGA
Mif1	GAACCGCAACTACAGTAAGCTGC	ACGTTGGCAGCGTTCATGTCGT
Mmp2	CATCGCCCATCATCAAGTTC	AAAGCATCATCCACGGTTTC
Pparg	AAGAGCTGACCCAATGGTTG	GCATCCTTCACAAGCATGAA
Psmd4	CCATCAACCAGCAGGAGTTTGG	CTGGCATCCATGTCAGCCGATT
Sgk1	CTCCCTAAACATCGTTTATAGAGAC	AAGTCAGTGAGGACGATGTG
Sod1	CCATCAGTATGGGGACAATACA	GGTCTCCAACATGCCTCTCT
Stat3	GTTCCTGGCACCTTGGATT	CAACGTGGCATGTGACTCTT
Tgfb1	TGATACGCCTGAGTGGCTGTCT	CACAAGAGCAGTGAGCGCTGAA
Timp1	GCAAAGAGCTTTCTCAAAGACC	AGGGATAGATAAACAGGGAAACACT
Timp3	CACGGAAGCCTCTGAAAGTC	TCCCACCTCTCCACAAAGTT
Tnfa	ATGGCCTCCCTCTCATCAGT	AAGGTACAACCCATCGGCTG
Tpb	GCTCTGGAATTGTACCGCAG	TGACTGCAGCAAATCGCTTG
Vegfa	ACTGGACCCTGGCTTTACTG	TCTGCTCTCCTTCTGTCGTG

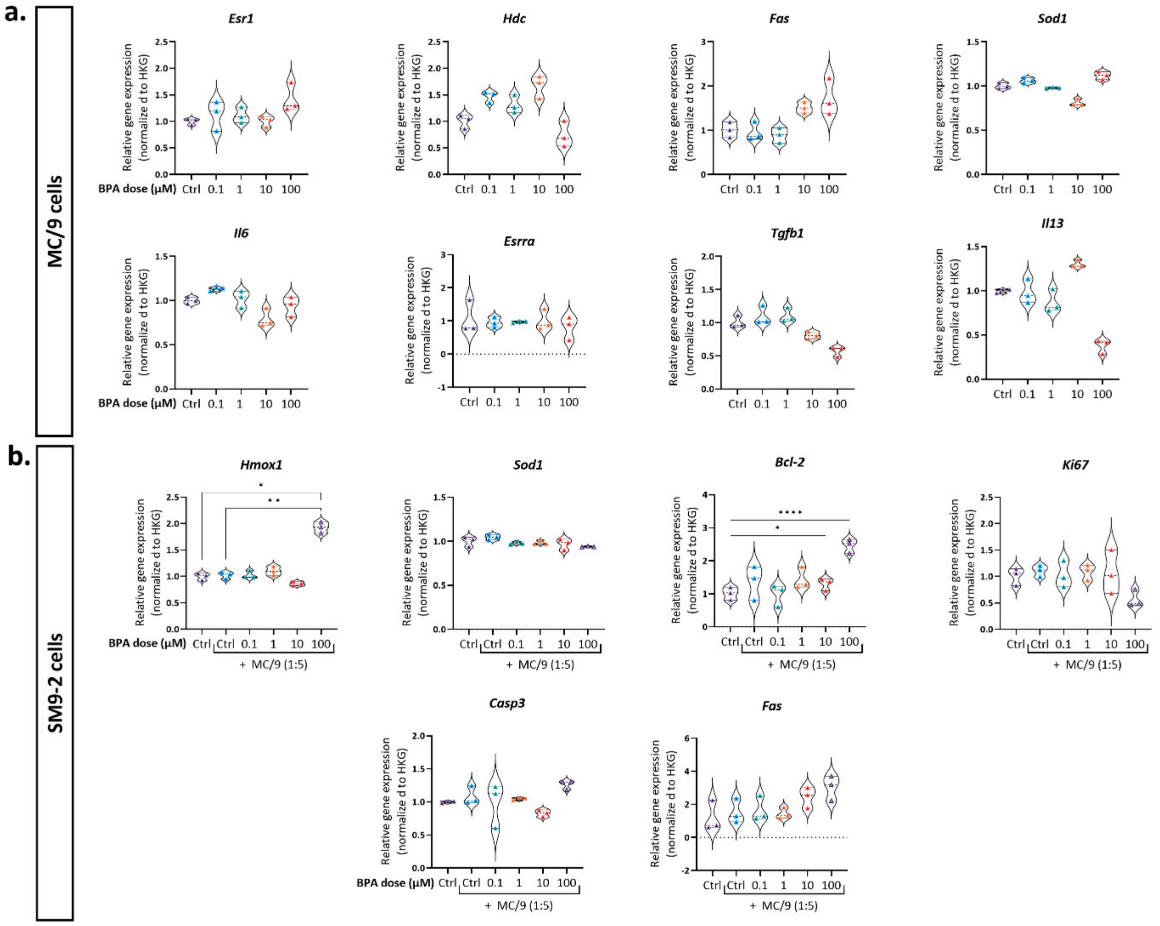

Figure S2. Additional results on BPA impact on the gene expression of MC/9 and SM9-2 cells following 24 h coculture. Violin plots show the results of qPCR analysis of total mRNA isolated from MC/9 (a) and SM9-2 (b) cells after 24 h exposure to BPA, investigating the expression of genes related to immune mediators and oxidative stress (*Mif1, Tgfb1, Il6, Il13, Hmox1, Sod1*), estrogen receptor (*Esr1, Essra*), mast cell activity (*Hdc*), and cell death (*Fas*, *Bcl-2, Casp3*) and proliferation (*Ki67*). Individual values represent the mean relative expression deriving from three technical replicates of single independent experiments (n=3), calculated as $2^{-\Delta\Delta Ct}$. Ct values were normalized to the geometric mean of the expression of two housekeeping genes (HKG; *Psmd4* and *Tbp*). Statistical analysis was performed using repetitive measures ANOVA, and all groups' means were compared to each other using Tukey's multiple comparison post hoc test. Statistical significance was defined as $p < 0.05$. p value: * = < 0.05; ** = < 0.001; **** = < 0.00001. HKG, housekeeping genes; BPA, Bisphenol A; *Esr1*, estrogen receptor alpha; *Hdc*, histidine decarboxylase; *Fas*, fas cell surface death receptor; *Sod1*, superoxide dismutase 1; *Il6*, interleukin 6; *Essra*, estrogen related receptor alpha; *Tgfb1*, transforming growth factor beta 1; *Il13*, interleukin 13; *Hmox1*, heme oxygenase 1; *Bcl-2*, apoptosis regulator B-cell lymphoma-2; *Casp3*, caspase 3; *Ki67*, antigen kiel 67; *Psmd4*, 26S proteasome non-ATPase regulatory subunit 4; *Tbp*, TATA box binding protein.
